# COVID-19 symptoms at time of testing and association with positivity among outpatients tested for SARS-CoV-2

**DOI:** 10.1371/journal.pone.0260879

**Published:** 2021-12-10

**Authors:** David A. Wohl, Amir H. Barzin, Sonia Napravnik, Thibaut Davy-Mendez, Jason R. Smedberg, Cecilia M. Thompson, Laura Ruegsegger, Matt Gilleskie, David J. Weber, Herbert C. Whinna, Melissa B. Miller

**Affiliations:** 1 Institute of Global Health and Infectious Diseases, The University of North Carolina at Chapel Hill, Chapel Hill, NC, United States of America; 2 Department of Family Medicine, The University of North Carolina at Chapel Hill, Chapel Hill, NC, United States of America; 3 Department of Psychiatry and Behavioral Sciences, University of California, San Francisco, CA, United States of America; 4 Clinical Microbiology Laboratory, McLendon Clinical Laboratories, The University of North Carolina Medical Center, Chapel Hill, NC, United States of America; 5 Department of Pathology and Laboratory Medicine, The University of North Carolina at Chapel Hill, Chapel Hill, NC, United States of America; Istanbul University Istanbul Faculty of Medicine: Istanbul Universitesi Istanbul Tip Fakultesi, TURKEY

## Abstract

**Introduction:**

Symptoms associated with SARS-CoV-2 infection remain incompletely understood, especially among ambulatory, non-hospitalized individuals. With host factors, symptoms predictive of SARS-CoV-2 could be used to guide testing and intervention strategies.

**Methods:**

Between March 16 and September 3, 2020, we examined the characteristics and symptoms reported by individuals presenting to a large outpatient testing program in the Southeastern US for nasopharyngeal SARS-CoV-2 RNA RT-PCR testing. Using self-reported symptoms, demographic characteristics, and exposure and travel histories, we identified the variables associated with testing positive using modified Poisson regression.

**Results:**

Among 20,177 tested individuals, the proportion positive was 9.4% (95% CI, 9.0–9.8) and was higher for men, younger individuals, and racial/ethnic minorities (all P<0.05); the positivity proportion was higher for Hispanics (26.9%; 95% CI. 24.9–29.0) compared to Blacks (8.6%; 95% CI, 7.6–9.7) or Whites (5.8%; 95% CI, 5.4–6.3). Individuals reporting contact with a COVID-19 case had the highest positivity proportion (22.8%; 95% CI, 21.5–24.1). Among the subset of 8,522 symptomatic adults who presented for testing after May 1, when complete symptom assessments were performed, SARS-CoV-2 RNA PCR was detected in 1,116 (13.1%). Of the reported symptoms, loss of taste or smell was most strongly associated with SARS-CoV-2 RNA detection with an adjusted risk ratio of 3.88 (95% CI, 3.46–4.35). The presence of chills, fever, cough, aches, headache, fatigue and nasal congestion also significantly increased the risk of detecting SARS-CoV-2 RNA, while diarrhea or nausea/vomiting, although not uncommon, were significantly more common in those with a negative test result. Symptom combinations were frequent with 67.9% experiencing ≥4 symptoms, including 19.8% with ≥8 symptoms; report of greater than three symptoms increased the risk of SARS-CoV-2 RNA detection.

**Conclusions:**

In a large outpatient population in the Southeastern US, several symptoms, most notably loss of taste or smell, and greater symptom burden were associated with detection of SARS-CoV-2 RNA. Persons of color and those with who were a contact of a COVID-19 case were also more likely to test positive. These findings suggest that, given limited SARS-CoV-2 testing capacity, symptom presentation and host characteristics can be used to guide testing and intervention prioritization.

## Introduction

As SARS-CoV-2 infection has rapidly spread across the globe to become a pandemic, an evolving picture has emerged of the clinical presentation of SARS-CoV-2 and the illness it causes, COVID-19 [1–3]. Initial reports among hospitalized patients described a predominately lower respiratory tract illness with fever, cough and dyspnea as common presenting symptoms [4–9]. Over time, a greater diversity of symptoms among those presenting with COVID-19 have been described, including gastrointestinal complaints, and loss of taste and smell [10–13].

Although the vast majority of those infected with SARS-CoV-2 remain outpatients, most descriptions of COVID-19 symptomatology have relied on patients requiring hospital admission [14–16]. There are also limited data on symptoms that are present early in disease course, including at time of testing [17]. Additionally, few reports of the clinical presentation of COVID-19 among outpatients have come from the US, a nation currently with the highest number of COVID-19 cases [18].

Although screening for SARS-CoV-2 infection in the US has dramatically increased, testing availability remains unable to meet demand [19]. Identification of factors associated with SARS-CoV-2 detection can be useful when devising screening prioritization strategies. Therefore, we sought to examine early COVID-19 symptomatology and host characteristics associated with SARS-CoV-2 detection among over 20,000 individuals presenting for outpatient SARS-CoV-2 testing at a large outpatient drive-through testing center located in the Southeastern US from March 16 through September 3, 2020.

## Methods

### Participants and setting

University of North Carolina Health Care (UNC Health) established a Respiratory Diagnostic Center (RDC) for outpatient SARS-CoV-2 screening on March 16, 2020 using a drive-through model. To be tested, individuals had to have at least one of the following symptoms: fever, chills, muscle aches, headache, cough, nasal congestion, sore throat, shortness of breath, nausea or vomiting, diarrhea, or abdominal pain. Loss of taste or smell and fatigue were added April 20 and May 1, respectively. Beginning May 1, asymptomatic individuals with scheduled procedures (e.g., endoscopy, chemotherapy) and surgeries at UNC Medical Center in Chapel Hill were also tested. On July 6, testing criteria were further broadened to include asymptomatic individuals with preexisting medical conditions, close contacts of confirmed cases, and individuals from historically marginalized populations disproportionately impacted by COVID-19. The study protocol was approved and granted a waiver of consent by the UNC School of Medicine Institutional Review Board.

### SARS-CoV-2 testing

Testing for SARS-CoV-2 RNA was the outcome of interest and was performed on a nasopharyngeal (NP) swab collected by staff trained in proper collection techniques. Flocked nylon swabs were used for specimen collection, immediately placed into universal transport media or phosphate buffered saline and transported at 2–8°C to UNC Medical Center’s Clinical Microbiology Laboratory for analysis. Real time reverse transcription (RT) PCR was conducted using one of the following Emergency Use Authorization tests: UNC Health SARS-CoV-2 real-time RT-PCR test, Abbott RealTime SARS-CoV-2 assay or Abbott Alinity m SARS-CoV-2 assay (both have a lower limit of detection of 100 copies/mL). As SARS-CoV-2 testing was initiated during the end of the influenza season, from March 16 to April 13, 2020 NP swab specimens were first tested with a respiratory panel that included at least influenza A and B and respiratory syncytial virus. Failure to detect other respiratory viruses led to reflex testing for SARS-CoV-2. After April 13, all patients had testing only for SARS-CoV-2.

### Measures

Prior to NP swab collection, individuals were asked to complete a written brief symptom and medical status assessment form, available in English and Spanish. The symptoms included in the assessment were those listed by the U.S. Centers for Disease Control and Prevention (CDC) as being associated with COVID-19, and was regularly updated, as described above [20]. The assessment also asked respondents to indicate the presence of one or more medical conditions considered to place an individual at greater risk of progression to severe COVID-19. The conditions included were those the CDC has identified as increasing the risk of severe complications of influenza [21], with the addition of hypertension [22]. All data used in the analyses were obtained from the UNC electronic health record, including demographics (self-reported date of birth, sex and race/ethnicity), SARS-CoV-2 test results, and RDC assessment responses.

### Statistical analyses

The RDC performed 22,336 SARS-CoV-2 tests from March 16 to September 3, with 90.3%, 8.2% and 1.5% of individuals tested once, twice and three or more times, respectively. Among individuals tested more than once, we included either the first positive test among those who tested positive, or the first test otherwise. Among 20,177 unique individuals tested, we estimated SARS-CoV-2 positivity proportion (expressed as percentage) and 95% confidence intervals (CI) by demographic and clinical characteristics. Longitudinal positivity proportions and 95% CI were plotted using centered sixth order polynomial regression.

To assess SARS-CoV-2 positivity by symptom and number of symptoms reported we further restricted the study population by excluding tests performed prior to May 1 (date all symptoms were included on RDC assessment), testing as part of scheduled procedures and surgeries at UNC (n = 3,071), and being asymptomatic (S1 Fig). Also excluded were children (<18 years old) given the relatively small number of tests done in this population and evidence that symptoms experienced by children differ from those experienced by adults [23]. Among these 8,522 symptomatic adults tested between May 1 and September 3, we calculated the proportion positive and 95% CI overall and stratified by symptom. We estimated unadjusted and adjusted risk ratios (prevalence ratios) and 95% CIs for the association between each symptom and SARS-CoV-2 positivity using robust (modified) Poisson regression, with multivariable analyses adjusted for sex, age and race/ethnicity.

In a subgroup further restricting the above 8,522 symptomatic adults to those who tested SARS-CoV-2 positive (n = 1,116) we compared symptom frequencies and number of symptoms reported by sex and age using Pearson’s chi-squared and Mann Whitney U tests, as indicated. To evaluate symptom combinations, we assessed co-occurrence of individual symptoms and created symptom categories including systemic (fever, chills, muscle aches, fatigue and headache), respiratory tract infection (URTI; nasal congestion, sore throat and cough), and gastrointestinal (GI; nausea or vomiting, abdominal pain and diarrhea). If an individual experienced at least one symptom within a symptom category they were assigned as experiencing that category. To further quantify the relationships between individual symptoms and symptom categories we calculated the Phi coefficient as a measure of association, comparable to the Pearson correlation coefficient for two binary factors. Analyses were performed using R 3.6.3 (Vienna, Austria), SAS 9.4 (Cary, NC) and GraphPad Prism 8.4.3 (San Diego, CA). All tests were two-sided and a P-value <0.05 was considered statistically significant.

## Results

### Characteristics of those tested and trends in testing

Overall, among 20,177 individuals with a SARS-CoV-2 RT-PCR test performed at the UNC RDC from March 16 to September 3, 2020, 61% were women, 64% White, 17% Black, 11% Hispanic and 8% of other and unknown race/ethnicity (other race includes Asian, American Indian or Alaska Native, and Native Hawaiian or Other Pacific Islander) (Table 1). The median age was 39 years (interquartile range [IQR, 25–56). Forty-seven percent of individuals did not report any exposure, 24% reported being a healthcare provider (among whom 10% also reported travel), 22% reported contact with a case (among whom 18% also reported travel), and 7% reported travel as the only potential exposure. The most frequently reported comorbidities were hypertension (16%), chronic lung disease (12%), immunodeficiency disorder (7%), diabetes (7%) and morbid obesity (6%), with each of the remaining conditions reported by fewer than 3% of those tested.

**Table 1:** Characteristics of 20,177 symptomatic and asymptomatic individuals tested for SARS-CoV-2, UNC RDC March 16 to September 3, 2020.

	SARS-CoV-2 (No.)		SARS-CoV-2 positivity % (95% CI)	P-Value
Characteristic ^a^	Not detected	Detected		
Total	18274	1903	9.4 (9.0–9.8)	
Sex				<0.001
Female	11355	1003	8.1 (7.6–8.6)	
Male	6904	896	11.5 (10.8–12.2)	
Race / ethnicity ^b^				<0.001
White	10311	639	5.8 (5.4–6.3)	
Black	2664	250	8.6 (7.6–9.7)	
Hispanic	1359	501	26.9 (24.9–29.0)	
Other	1262	100	7.3 (6.0–8.9)	
Age, years				<0.001
≤17	1648	199	10.8 (9.4–12.2)	
18–22	1869	572	23.4 (21.8–25.1)	
23–59	10988	959	8.0 (7.5–8.5)	
≥60	3769	173	4.4 (3.7–5.0)	
Comorbidities / Conditions ^c^				
High blood pressure	2168	171	7.3 (6.3–8.4)	<0.001
Chronic lung disease	1680	117	6.5 (5.4–7.8)	<0.001
Immunodeficiency disorder	970	58	5.6 (4.3–7.2)	<0.001
Diabetes	936	100	9.7 (7.9–11.6)	0.35
Morbid obesity	808	82	9.2 (7.4–11.3)	0.12
Cardiovascular disease	453	23	4.8 (3.1–7.2)	<0.001
Chronic kidney disease	212	13	5.8 (3.1, 9.7)	0.02
Chronic liver disease	97	5	4.9 (1.6–11.1)	0.07
Chronic blood disorder	99	7	6.6 (2.7–13.1)	0.21
Neurological disorder	186	6	3.1 (1.2–6.7)	<0.001
Pregnancy	186	23	11.0 (6.8–15.3)	0.27
Smoking				<0.001
Current	854	68	7.4 (5.7–9.1)	
Past	1570	109	6.5 (5.3–7.7)	
Never	9621	1346	12.3 (11.7, 12.9)	
Exposure (past 14 days) ^d^				<0.001
Healthcare personnel	4140	270	6.1 (5.4, 6.8)	
Case contact	3150	929	22.8 (21.5, 24.1)	
Travel outside of NC	1113	92	7.6 (6.1, 9.1)	
No exposure reported	8269	410	4.7 (4.3, 5.2)	
SARS-CoV-2 Test Date				<0.001
March 16–May 17	3402	214	5.9 (5.2–6.7)	
May 18–July 26	6279	911	12.7 (11.9–13.5)	
July 27–September 3	8593	778	8.3 (7.8–8.9)	

Abbreviations: UNC, University of North Carolina; RDC, Respiratory Diagnostic Center; No., number; SARS-CoV-2, severe acute respiratory coronavirus 2; CI, Confidence Interval.

^a^ Characteristic N: sex (n = 20158), race/ethnicity (n = 17086), age (n = 20177), chronic lung disease (n = 15292), diabetes (n = 15290), high blood pressure (14845), cardiovascular disease (n = 15290), chronic kidney disease (n = 15289), chronic liver disease (n = 15287), chronic blood disorder (n = 14820), weak immune system (n = 14810), neurologic disorder (n = 14794), pregnancy (n = 9380 females), morbid obesity (n = 14763), current smoker (n = 13564), ever smoker (n = 13568), travel outside NC (n = 18395), case contact (n = 18359), health care worker (n = 18392).

^b^ Other race includes Asian, American Indian or Alaska Native, and Native Hawaiian or Other Pacific Islander.

^c^ P-values for comorbidities/conditions represent a contrast between having a specific comorbidity or condition and not having that specific comorbidity or condition.

^d^ Exposure assigned hierarchically in order as health care worker, case contact and travel.

**Fig 1:**
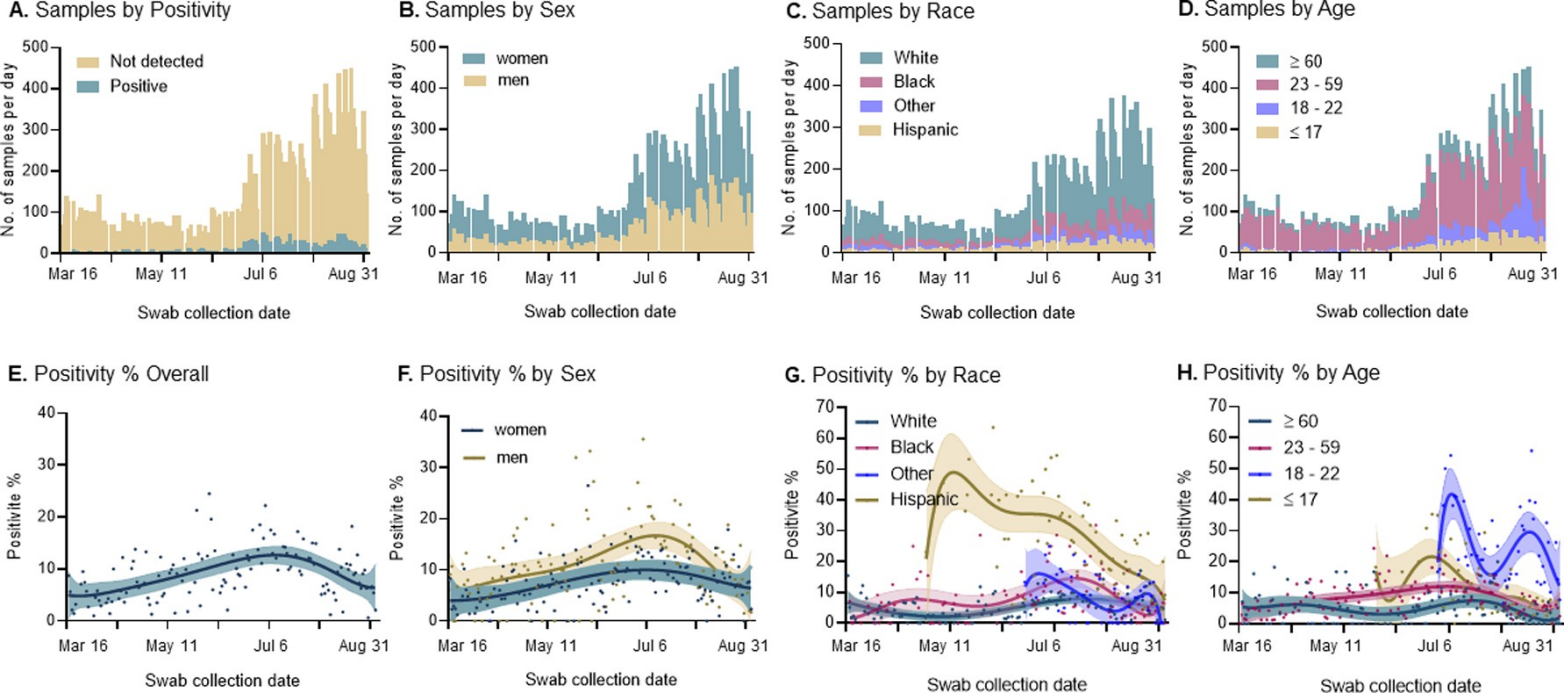
SARS-CoV-2 testing and positivity proportion among 20,177 symptomatic and asymptomatic individuals, UNC RDC March 16 to September 3, 2020. Number of individuals tested per day stratified by **(A)** SARS-CoV-2 positive result, **(B)** sex, **(C)** race/ethnicity and **(D)** age. Longitudinal SARS-CoV-2 positivity proportions **(E)** overall and stratified by **(F)** sex, **(G)** race/ethnicity and **(H)** age, with plotted centered sixth order polynomial regression and 95% confidence intervals. Abbreviations: UNC, University of North Carolina; RDC, Respiratory Diagnostic Center; No., number; SARS-CoV-2, severe acute respiratory coronavirus 2.

The number of tests performed per day increased during the study period, reflecting increases in testing capacity and changes in testing guidelines (Fig 1A). Specifically, a large increase in tests was observed starting July 6, coincident with expanded laboratory testing capacity, broadening of the testing criteria, and the addition of testing of patients prior to scheduled surgery or certain procedures (e.g., endoscopy). A later rise in daily number of tests performed was observed at the beginning of August when the academic year began at UNC-Chapel Hill and students returned to campus. A greater number of women than men and of White individuals than other race/ethnicity groups were tested throughout the study period (Fig 1B and 1C). The number of individuals 18 to 22 years of age increased over time, as expected with university campus opening (Fig 1D).

### Test positivity

Overall, SARS-COV-2 positivity among all those tested including symptomatic and asymptomatic individuals was 9.4% (95% Confidence Interval [CI], 9.0–9.8), varying over calendar time (Table 1 and Fig 1E). The lowest weekly positive proportion was the first week of testing, March 16 to 22, with a positivity of 3.1% (95% CI, 1.7–5.1), rising to its highest level the week of June 29 to July 5 with a positivity of 15.9% (95% CI, 13.6–18.4), and with a most recent positivity proportion during August 31 to September 3 of 5.3% (95% CI, 3.8–7.2). Overall, the positivity proportion was higher among men, non-White individuals, and younger individuals, especially those 18 to 22 years of age, with differences by sex and race/ethnicity decreasing over calendar time (Fig 1F–1H). The positivity proportion was considerably higher for Hispanic individuals at 26.9% (95% CI, 24.9–29.0) compared to those who were Black at 8.6% (95% CI, 7.6–9.7) or White at 5.8% (95% CI, 5.4–6.3). Individuals with comorbidities were in general equally or less likely to test positive, whereas individuals reporting contact with a case had the highest positivity proportion (22.8%, 95% CI, 21.5–24.1).

### Symptom presentation among those tested

**Fig 2:**
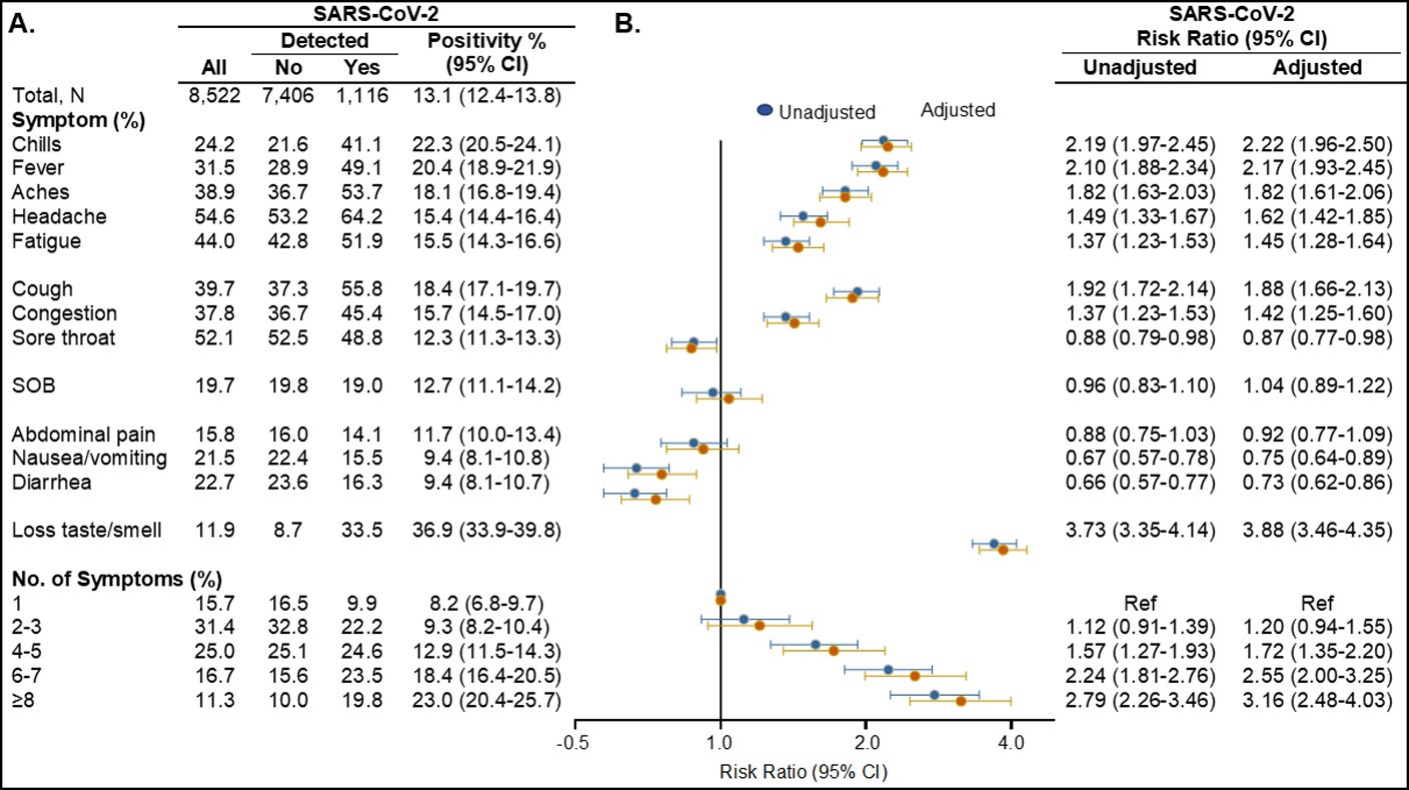
SARS-CoV-2 positivity by COVID-19 symptom among 8,522 symptomatic adults, UNC RDC May 1 to September 3, 2020. **(A)** Percent of individuals reporting COVID-19 symptom and positivity proportion with 95% confidence interval for each COVID-19 symptom. **(B)** Unadjusted and adjusted risk ratios and 95% confidence intervals for testing positive for each COVID-19 symptom, with adjusted estimates adjusted for sex, age and race/ethnicity. The unadjusted and adjusted risk ratios for each symptom contrast the presence of that symptom with not having that symptom. Abbreviations: UNC, University of North Carolina; RDC, Respiratory Diagnostic Center; No., number; SARS-CoV-2, severe acute respiratory coronavirus 2; CI, Confidence Interval; SOB, shortness of breath.

Among 11,317 adults tested between May 1 and September 3, 24.7% (n = 2,795) did not report any symptoms, with 183 of these testing SARS-CoV-2 RNA positive (6.6%). Among adults reporting at least one symptom (n = 8,522), the overall SARS-COV-2 positivity was 13.1% (95% CI 12.4–13.8). Individuals reported symptom onset a median of 3 days prior to testing (IQR 2–5), comparable across symptoms. The most frequently reported symptoms were headache (54.6%), sore throat (52.1%) and fatigue (44.0%) (Fig 2A).

The symptoms associated with the highest positivity proportion included new onset of loss of taste and/or smell, chills, and fever, with relative risks of testing positive of 3.73, 2.19 and 2.10, respectively (Fig 2B). In adjusted analyses the risk ratio (RR) of testing positive comparing those with and without a new loss of taste or smell was 3.88 (95% CI, 3.46–4.35). Experiencing chills and fever were also each related to over twice the risk of testing positive in adjusted analyses (RR = 2.22 comparing presence of chills to not having chills; 95% CI, 1.96–2.50, and RR = 2.17 for presence of fever versus not having a fever, 95% CI, 1.93–2.45). Notably, over a third of those with detectable SARS-CoV-2 RNA reported loss of tase or smell (36.9%; 95% CI, 33.9–39.8), and 22.3% and 20.4% reported chills or fever tested positive (95% CI, 20.5–24.1 and 18.9–21.9, respectively). Reporting more than one symptom was common (84.3%), with over one-half of individuals reporting 4 or more symptoms (53.0%). Experiencing a greater number of symptoms was strongly related to the probability of testing positive, with 8.2% testing positive among those reporting one symptom, 9.3% positive for 2–3 symptoms, 12.9% positive for 4–5 symptoms, 18.4% positive for 6–7 symptoms, and 23.0% for 8 or more symptoms.

### Symptom presentation among those with detectable SARS-CoV-2 RNA

**Fig 3:**
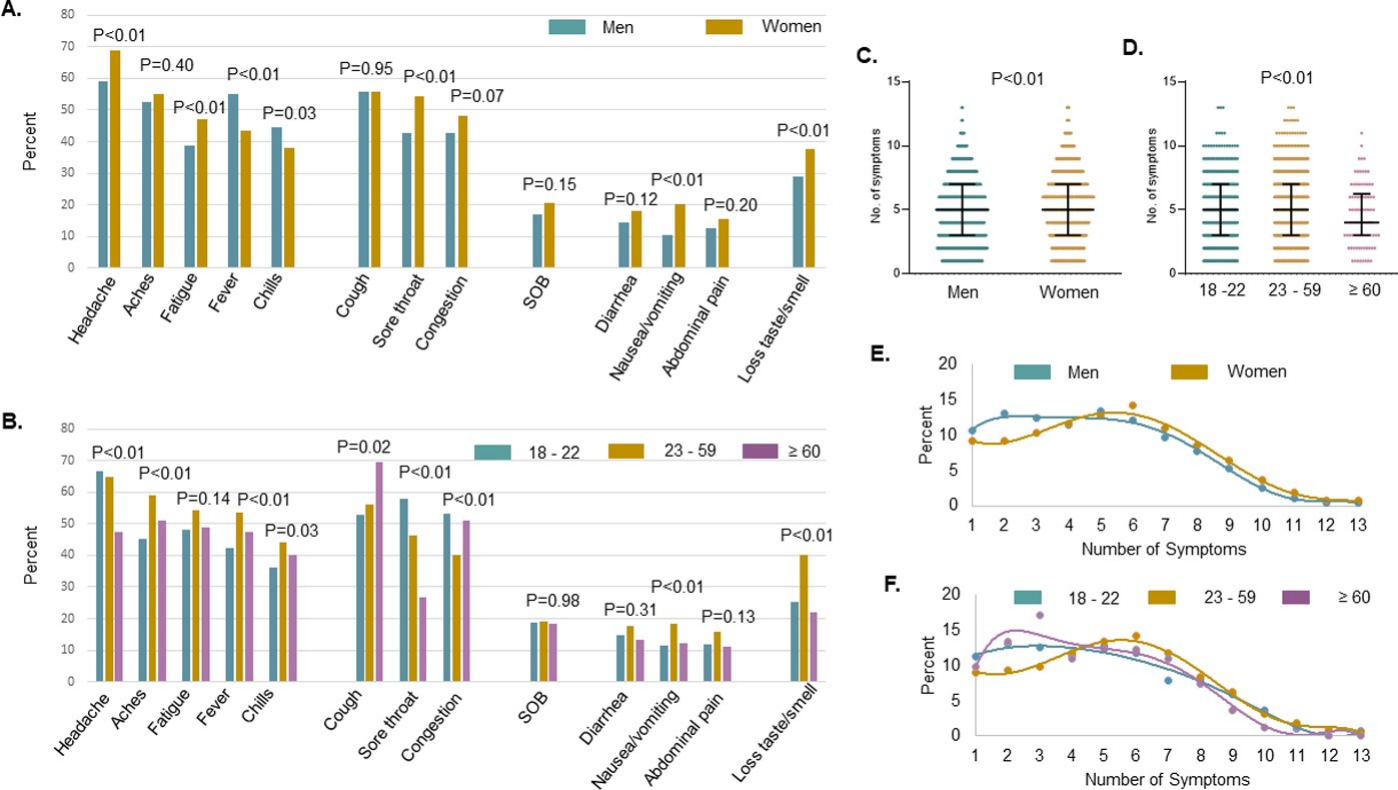
COVID-19 symptoms among 1,116 symptomatic adults with positive SARS-CoV-2 RNA, UNC RDC May 1 to September 3, 2020. **(A and B)** Percent of individuals with COVID-19 symptom by sex and age. **(C and D)** Box plots of number of symptoms by sex and age. **(E and F)** Percent of individuals with number symptoms with plotted centered sixth order polynomial regression, by sex and age. Abbreviations: UNC, University of North Carolina; RDC, Respiratory Diagnostic Center; No., number; SARS-CoV-2, severe acute respiratory coronavirus 2; SOB, shortness of breath.

Among the 1,116 symptomatic adults who were SARS-CoV-2 RNA positive, the most commonly reported symptoms were headache (64.2%), cough (55.8%), muscle or body aches (53.7%), fatigue (51.9%), sore throat (48.8%) and fever (49.1%) (Fig 2A). Women were more likely to report certain symptoms, particularly headache, as well as fatigue, sore throat, nausea or vomiting, and loss of taste and/or smell, whereas men were more likely to report fever (Fig 3A, all P<0.01). Individuals at least 60 years of age were more likely to report cough and nasal congestion than younger individuals, whereas individuals 23 to 59 years of age were more likely to report muscle or body aches, fever, chills, nausea or vomiting and loss of taste and/or smell in comparison to both younger and older individuals (Fig 3B, all P<0.05). Overall, the median number of symptoms was comparable by sex with a median of 5 symptoms reported by both men and women (IQR 3–7) (Fig 3C). However, women were more likely to experience a greater number of symptoms overall than men (Fig 3C and 3E, both P<0.01), with 47.0% versus 39.2% of women and men, respectively, reporting 6 or more symptoms (P<0.01). Individuals 23 to 59 years of age experienced more symptoms than those 18 to 22 years-old, with those at least 60 years of age reporting on average fewer symptoms (Fig 3D and 3F, P<0.01).

**Fig 4:**
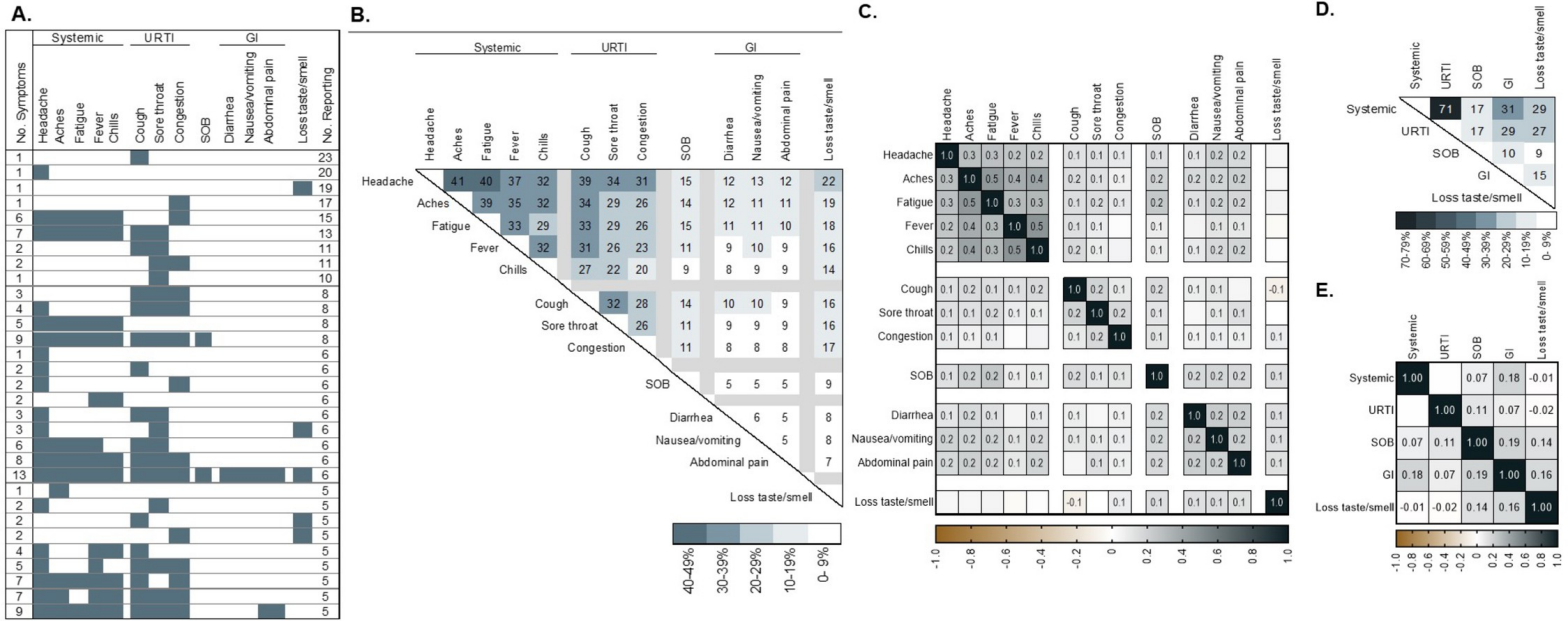
COVID-19 symptom combinations among 1,116 symptomatic outpatient adults with positive SARS-CoV-2 RNA, UNC RDC May 1 to September 3, 2020. **(A)** All symptom combinations reported by at least five individuals. **(B)** Percent reporting symptom combinations. **(C)** Phi correlation heat map for symptom combinations. **(D)** Percent reporting symptom category combinations. **(E)** Phi correlation heat map for symptom category combinations. Abbreviations: UNC, University of North Carolina; RDC, Respiratory Diagnostic Center; No., number; SARS-CoV-2, severe acute respiratory coronavirus 2; SOB, shortness of breath; URTI, upper respiratory tract infection; GI, gastrointestinal.

The 1,116 symptomatic SARS-CoV-2 RNA positive adults reported 662 unique symptom combinations, with 31 combinations experienced by at least 5 individuals (total n = 270, 24.2%) (Fig 4A). An additional 18 unique combinations were each reported by 4 individuals (n = 72, 6.5%), 37 combinations by 3 individuals (n = 111, 9.9%), 87 combinations by 2 individuals (n = 174, 15.6%), and 489 combinations each reported by 1 individual (n = 489, 43.8%). The most frequently reported dual symptom combinations were systemic symptoms with headache and aches, headache and fatigue, and aches and fatigue (reported by 41, 40 and 39%, respectively) (Fig 4B). URTI symptoms also commonly co-occurred with 32, 28 and 26% reporting cough and sore throat, cough and nasal congestion, and sore throat and nasal congestion, respectively. The highest Phi coefficient of 0.5 was observed for aches and fatigue, and fever and chills, indicating a strong positive association between these two symptoms (Fig 4C). For example, 75% versus 30% experienced aches, comparing those experiencing and not experiencing fatigue.

The most frequently co-occurring symptom categories included systemic symptoms along with URTI, gastrointestinal and loss of taste and/or smell, experienced by 71, 31 and 29%, respectively (Fig 4D). Symptom categories were at most weakly associated with each other, with all Phi coefficients <0.20 (Fig 4E). The strongest association observed was between shortness of breath and GI symptoms (Phi coefficient = 0.19), with shortness of breath reported by 29% versus 51% contrasting those with and without a GI symptom. Systemic and URTI symptoms, although frequently co-occurring, were not associated with each other (Phi coefficient = 0.004), with systemic symptoms reported by 85% and 86% among both those with and without an URTI symptom, respectively.

## Discussion

Among a large number of US outpatients tested for SARS-CoV-2 we identified several symptoms and individual characteristics associated with test positivity that can be used to inform predictive risk assessments. As expected, loss of taste or smell, chills, fever, cough, aches, headache, fatigue and nasal congestion were associated with positive test for SARS-CoV-2. In contrast, gastrointestinal symptoms of diarrhea or nausea/vomiting, were significantly more common in those with a negative test result. Experiencing a greater number of symptoms was also associated with testing positive for SARS-CoV-2. Loss of taste or smell is well-appreciated to be associated with COVID-19 [12, 13, 24] and we found that over one-third of individuals with loss of taste or smell tested positive. Individuals experiencing a new loss of taste or smell were almost 4 times as likely to be SARS-CoV-2 positive (adjusted risk ratio 3.88 95% CI 3.46–4.35).

Among SARS-CoV-2 RNA positive, symptomatic adults, less specific symptoms were also commonly reported with over one-half experiencing headache, cough, muscle and body aches and fatigue. These findings are consistent with other studies from the US among outpatients and healthcare personnel [25–27]. The burden of symptoms experienced among outpatients with recent onset of COVID-19 symptoms (median 3 days) differs from that reported among hospitalized individuals, with a recent global meta-analysis including primarily studies among hospitalized individuals identifying fever as the most frequently reported symptom at 78% [28]. Conversely, while gastrointestinal symptoms were not uncommon among those testing SARS-CoV-2 RNA positive, with diarrhea and nausea/vomiting reported by 16.3% and 15.5% respectively; diarrhea and nausea/vomiting were significantly more commonly reported by those who tested negative for SARS-CoV-2 infection (23.6% and 22.4%, respectively), even after adjustment for age, sex, and race.

We found that there were differences in reported symptoms patterns among those testing SARS-CoV-2 positive based on gender and age. Systemic symptoms were, in general, more likely to be reported by individuals 23 to 59 years of age and women were much more likely to report headaches and fatigue, whereas men were more likely to report fever and chills. Older individuals experienced a cough and congestion more frequently, and younger individuals were more likely to report a sore throat. Notably, shortness of breath was not associated with SARS-CoV-2 infection but was reported by 19% of those who tested positive.

Experiencing a greater number of symptoms increased the likelihood of a positive SARS-CoV-2 test, consistent with other reports [26, 27]. Among those infected, more than two-thirds (67.9%) reported four or more symptoms, including almost 20% reporting eight or more symptoms. A vast diversity of symptom combinations was reported among symptomatic adults SARS-CoV-2 RNA positive, with combinations of systemic symptoms being the most common, followed by combinations of URTI symptoms. Seven out of 10 individuals with COVID-19 experienced both a systemic and an URTI symptom. Approximately 3 in 10 experienced a systemic and gastrointestinal symptom, a systemic symptom and loss of taste and/or smell, an URTI and gastrointestinal symptom, and an URTI symptom and loss of taste and/or smell. Although there was no association detected between systemic symptoms and loss of taste or smell, a weak positive relationship was observed between loss of taste or smell and shortness of breath and gastrointestinal symptoms.

In addition to symptoms and symptom complexes, individual characteristics were also associated with testing positive for SARS-CoV-2 infection. In particular, the positivity proportion was dramatically greater for those reporting Hispanic ethnicity and was approximately 3-fold higher than for Black individuals and 5-fold higher than for Whites. While testing criteria in North Carolina expanded to include those from underserved populations during the analysis period [29], this was the result of the recognition of evolving disparities in the state, as in most of the US, with COVID-19 unequally impacting persons of color in both rates of infection and mortality [30]. A number of explanations for these disparities have been posited including the greater proportion of essential workers who are people of color, lack of personal protective equipment at work places, denser living conditions especially for agricultural workers, prevalent comorbid conditions, barriers to accessing healthcare, and poverty [31–33]. The RDC was located within an hour drive of meat processing plants and large farms where the vast majority of the employees are Hispanic. While the assessment (available in Spanish) did not ask about occupation or occupational exposures, it is likely that many of those self-described as being Hispanic work in these nearby industries.

We also observed differences across time in the proportion testing positive, corresponding to changes in testing guidelines. However, consistently the groups at highest risk of testing positive were racial and ethnic minorities, especially individuals identifying as Hispanic, young adults aged 18 to 22 years of age, and individuals with an exposure to a known case (26.9%, 23.4% and 22.8% positive, respectively). Although the proportion testing positive decreased towards the end of August with less stringent criteria on who was eligible to be tested and expanded pre-operative and procedure testing, the SARS-CoV-2 positive proportion remained high at over 5%.

There are several potential practical applications of the identification of symptom and host characteristics associated with testing positive for SARS-CoV-2, especially when testing demand outstrips testing supply. Foremost, identifying symptoms and patient characteristics associated with SARS-CoV-2 infection can inform the development of predictive risk tools that could be used to prioritize testing to those most likely to be infected followed by the institution of public health measures such as isolation, case investigation and contact tracing to prevent further transmission. When testing resources are scarce, a high probability for infection could lead to assumption of positivity. Predictive tools can also assist in triage and prioritization of individuals for therapeutic interventions, such as infused monoclonal antibodies, which may be used for prophylaxis (pre- and post-exposure) to prevent infection and treatment to prevent disease progression.

Our ability to identify potential predictors of testing positive for SARS-CoV-2 is strengthened by the analysis of a large number of individuals with and without symptoms who were tested using NP swab, which is a gold standard for SARS-CoV-2 infection diagnosis. Swabbing was conducted uniformly by trained staff, and the PCR assay by a single laboratory. We assessed symptoms using a standardized instrument at the time of testing. Additionally, the symptom survey was available in English and Spanish. However, there are also limitations that should also be considered when interpreting these findings. As with any diagnostic test, the SARS-CoV-2 assays used may have misclassified individuals, especially individuals with lower viral loads [34–38]. SARS-CoV-2 shedding in the upper respiratory tract begins a couple of days prior to symptom onset and may be highest in the first week of infection during which symptoms may still be mild. Additionally, our study population included individuals being tested for SARS-CoV-2 at one outpatient testing center located nearby a large tertiary care referral hospital in the Southeastern US; our findings may not be generalizable to all populations with SARS-CoV-2 infection in the US. However, our study of symptoms associated with early COVID-19 are more applicable to an ambulatory population than studies conducted among hospitalized patients or health care workers [8, 39, 40]. Further, the majority of the period of analysis was between influenza seasons. Symptoms of COVID-19 and influenza overlap and during times when influenza is spreading, a greater number of symptomatic individuals may present for testing for both infections. Additional analyses will be needed to identify distinguishing symptom patterns during seasons when both viruses are circulating.

In conclusion, we identified several symptoms including new onset of loss of taste and/or smell, chills, and fever that along with symptom number were associated with detection of SARS-CoV-2 RNA in outpatients. Host race and ethnicity were also associated with SARS-CoV-2 positivity, reflecting the demography of the pandemic. Given the ongoing spread of SARS-CoV-2 within and outside of the US and the limited capacity to provide sufficient testing, these findings can be used to inform strategies to identify those most likely to be infected with SARS-CoV-2 and allocate testing and direct preventative and therapeutic interventions.

## Supporting information

S1 Fig Flow diagram of individuals for SARS-CoV-2, University of North Carolina Respiratory Diagnostic Center, March 16 to September 3, 2020. (TIF)
